# The role of microRNAs in cardiovascular disease

**Published:** 2013

**Authors:** Sarah Sadat Aghabozorg Afjeh, Sayyed Mohammad Hossein Ghaderian

**Affiliations:** Department of Medical Genetics, Shahid Beheshti University of Medical Sciences, Tehran, Iran.

**Keywords:** microRNA, cardiovascular disease

## Abstract

Cardiovascular disease has become the main factor of death and birth defects in the world. There are some therapeutic structures and drugs for curative and palliative therapy of the disease, but to the aim of accessing reliable therapy or to postpone onset of disease, especially for individuals with heritable coronary artery disease in their pedigree Genetic engineering technologies are making advances in the field by identifying oligonucleotides with higher potencies which can be easily targeted against almost any gene, particularly interfering RNA (RNAi). Recently, the focus of RNAi approaches has encompassed the use of synthetic sequences to mimic or silence endogenous microRNAs (miRNAs) that are abruptly dysregulated following cardiovascular diseases. In this review, we summarize the role of miRNAs in heart development and vascular system as two main factors of birth defects and adult morbidity and mortality and miRNAS as new therapeutic agents.

Cardiovascular disease, including its most severe complication myocardial infarction, has become the main factor of death in the world (1, 2).

More than 80% of sudden cardiac deaths in the world are caused by atherosclerotic coronary artery disease (CAD), and the remaining 20% of cases are caused by other diseases including cardiomyopathies, congenital heart disease, left ventricular hypertrophy, aortic valve disease, and other cardiac disorders. The familial aggregation of coronary heart disease can be in large part accounted for by a clustering of cardiovascular disease risk factors separated by environmental risk factors and genetic risk factors. To elucidate the determinants of cardiovascular disease, many epidemiological studies have focused on the behavioral and lifestyle determinants of these risk factors, whereas, others have examined whether specific candidate genes influence quantitative variation in these phenotypes (3). There are two separated environmental factors which lead to the disease, including habitual factors like smoking or other lifestyles, and epidemiological factors or factors related with the place an individual lives like air pollution and other environmental factors. Many authors have examined the association of height, weight, body mass index (BMI), cholesterol level, and pulse rate with blood pressure phenotypes as environmental factors and all the traits have shown a significant association (4, 5). As mentioned before there are also genetic risk factors causing cardiovascular disease. Despite years of intensive research, not a single genetic risk factor is used for risk assessment. The new strategy of genome-wide association (GWA) studies (for example, see http://www.wtccc. org.uk/) is starting to reveal novel genetic factors that contribute to disease risk. The spectrum of the genetic variants that predispose to CVD spans from rare, highly deleterious mutations responsible for Mendelian diseases which are usually identified by linkage studies, to common polymorphisms (minor allele frequency >1%) that, alone or in combination, modulate the risk of the disease. Because complex diseases do not follow a clear pattern of Mendelian inheritance, the strategy used to identify their genes of predisposition is usually not based on family studies but on a radically different approach called “genetic association” analysis (6). Also, in the case of complex diseases such as myocardial infarction (MI), linkage analysis is compounded by genetic heterogeneity of this disease and many other factors, including incomplete penetrance of disease-causing genes interaction with environmental factors, the high prevalence of the disease-causing allele in the population, and late onset of disease (7). According to researches based on Meta-analysis technique published on 2011, specific genetic loci were introduced to be related with cardiovascular disease and more specifically with MI. Expression changes in some of them cause increasing risk of MI (8, 9).

Regarding these loci as important genetic risk factors for the disease, endogenous micro RNAs (miRNAs) can be considered as factors involved in cardiac disease by their role in regulating the genes expression.

Recently, the focus of interfering RNA (RNAi) approaches has encompassed the use of synthetic sequences to mimic or silence endogenous miRNAs that are abruptly dysregulated following cardiovascular diseases. The implications of miRNAs in the pathological process of the cardiovascular system have recently been recognized, and research on miRNAs in relation to cardiovascular disease has now become a rapidly evolving field (10).

**Biology of miRNA**

Epigenetic control mechanisms including DNA methylation, histone modification, and non coding RNAs as well as differential RNA splicing, play a key role in the regulation of tissue homeostasis and disease development and enable the cell to respond quickly to environmental changes (11, 12). Noncoding RNAs are functional RNA molecules that belong to several groups and are involved in many cellular processes. miRNAs are a class of short, ~22-nucleotide, double strand non-coding RNAs that are endogenously transcribed and control gene expression by either inducing mRNA degradation or blocking translation. The first evidence that double strand RNA (dsRNA) could achieve efficient gene silencing through miRNAs came from the studies of lineage mutant named lin-4, which affect developmental timing in the nematode C. elegans in 1993 (13). Further analyses in Drosophila melanogaster have contributed toward under-standing the biochemical nature of the miRNA pathway.

miRNA genes are transcribed by RNA polymerase II to yield pre-miRNA transcripts that are then processed by the nuclear enzymes Drosha (14). The resulting 70 mer transcript is folded into a stem loop structure that is then exported into the cytoplasm by the shuttling factor Exportin 5 (15). 

The cytoplasmic enzyme Dicer, a member of the class III RNA endonuclease, is then required to process pre-miRNAs into double-stranded mature ~19–25 nt, miRNA generating 2 nucleotide overhangs at the 3’ termini. These miRNAs are recognized and bound by a multi-protein complex called the RNA-induced silencing complex (RISC) (16).

Each miRNA duplex is formed by a guide strand and a passenger strand. An enzyme within the RISC, the endonuclease Argonaute 2 (Ago 2) catalyzes the unwinding of the miRNA duplex. Once unwound, the guide strand is incorporated into the RISC, while the passenger strand is released. RISC uses the guide strand to find the mRNA that has a complementary sequence leading to the endonucleolytic cleavage of the target miRNA (17).

**miRNAs in Cardiovascular Disease**

Recent studies have shown that miRNAs play an essential role in some biological processes such as cell proliferation or cell differentiation, and apoptosis. Also, they are associated with important diseases including cancer and cardiovascular diseases (18-20).

According to Sanger institute and miRNA databases (21, 22) more than 1500 miRNAs have been identified in humans, current databases report 1600 precursors and 2042 mature miR sequences for humans (22). Among them, a subset of miRNAs including miR-1, miR-133, miR-206 and miR-208 are muscle tissue-specific, and have been called *myomiRs *(23-26).

Also, there are many miRNAs which participate in cardiac related diseases and their changes can be considered as a biomarker for the disease. In this review, we summarize the role of miRs in heart development and vascular system as two main factors of birth defects and adult morbidity and mortality (27-30).

**miRNA and cardiac development**

Several accurate and complex interactions among variants of cell types from several lineages, cardiomyocytes, endocardial, epicardial and vascular cells, fibroblasts and cells of the conduction system, are required for heart formation. Many specific miRNAs are enriched in different cardiac cell types and were reported to participate in the specification of cell identity (31).

Some experiments with different approaches demonstrated the role of miRNAs during vertebrate development. One of these approaches and also the first evidence for a regulation of endothelial cell functions by miRs came from the studies in which the first and second exons of the Dicer, miRNA processing enzyme encoding gene, were deleted using creatine kinase (Cre) recombinase expressed under the control of endogenous Nkx2.5 (one of the earliest cardiac markers, during mouse and human embryonic stem cells differentiation) regulator elements, which have been reported to lead to embryonic lethality in mice at embryonic stage, between days 12.5 and 14.5 of gestation (32, 33), due to either a loss of pluripotent stem cells (34) or disrupt angiogenesis in the embryo (33). Also, adult mice without Dicer genes in the myocardium has been shown to have a high incidence of sudden death, cardiac hypertrophy and reactivation of the fetal cardiac gene program (35).

Moreover, blood vessel formation/maintenance in embryos and yolk sacs was severely compromised and the expression of VEGF, FLT1, KDR, and TIE1 was altered in the mutant embryos indicating an essential role of Dicer for embryonic blood vessel development (11, 33).

Rao *et al.* used muscle creatine kinase (MCK)-Cre mice system to perform studies on mice with a muscle-specific deletion of the DiGeorge syndrome critical region gene 8 (DGCR8), which is required for the production of all canonical miRs. Since endogenous MCK expression reportedly peaks around birth and declines to 40 % of peak levels by day 10, and the phenotypic outcome was similar to the cardiac-specific dicer deficient mice, it seems that miRNAs play an important role in maintaining cardiac function in mature cardio-myocytes (32).

Since there are different reports about the role of miRNAs in adult heart, further studies are required in this filed.

Muscle-specific mirRNAs miR-1 and miR-133, as conserved miRNAs which are derived from common precursor, play their role during development and in adults, and were among the first miRs that had been identified as major regulators of muscle lineage commitment and had critical role in regulating muscle proliferation and differentiation (11).

Studies based on deep sequencing of a small RNA library reported that miRNA (miR)-1 was quite enriched and accounted for nearly 40% of all known miRNAs in the adult heart (32). The miR-1 miRNA precursor is a small micro RNA that regulates its target protein's expression in the cell. In humans, there are two distinct miRNAs that share an identical mature sequence, these are called miR-1-1 and miR-1-2 which target the same sequences and it has also been shown that in miR-1-2-deficient mice with miR-1-1 expression, there are spectrum of abnormalities, including ventricular septal defects in a subset that suffer early lethality, cardiac rhythm disturbances in those that survive (32) and functional defects in regulation of cardiac morphogenesis, electrical conduction, and cell cycle control (36). Studies showed that miR-1 regulates cardiac differentiation (36-39) and controls heart development in mice by regulation of the cardiac transcription factor Hand2 (32).

Also, miR-1 is downregulated in myocardial infarcted tissue compared to healthy heart tissue and Plasma levels of miR-1 can be used as a sensitive biomarker for myocardial infarction (40).

Although miR-1 and miR-133 are co-transcribed, their function appears clearly distinct. miR-133a double mutant mice are normal, whereas, the deletion of both miR-133a genes causes late embryonic or neonatal lethality due to ventricular septal defects in approximately half of the double-mutant embryos or neonates and abnormalities in cardiomyocyte proliferation (32, 41).

Caré et al, found that the expression of miR-133, which was transcribed together with miR-1 as a bicistronic cluster, was decreased in the left ventricle of the above mentioned three hypertrophic models (42). Mice lacking either miR-133a-1 or miR-133a-2 that survive to adult, suffer from dilated cardiomyopathy and succumb to heart failure.

These experiments prove the important role of miRNAs for the development of the organ. The heart as a particularly informative model for such organ patterning, has numerous transcriptional networks that establish chamber-specific gene expression and function (32, 43).

**miRNAs in Vascular system and angiogenesis**

The vascular system is fundamental for embryonic development and adult life, and aberrant vascularization is associated with numerous diseases, including cancer, atherosclerosis and stroke. Vascular system needs the establishment and remodeling of a contiguous series of lumenized tubes made of endothelial cells for its formation and function (31).

New studies indicated that miRNAs are highly expressed in vasculature and are critical modulators for vascular cell differentiation, contraction, migration, apoptosis, and dysregulation of their expression can cause vessel diseases (44).

Recently, a few specific miRNAs that regulate endothelial cell functions and angiogenesis have been described (31) which are described.

One of the new studied miRNAs in this field is miR-126 which is known as an angiogenesis regulator in development and neoangiogenesis after myocardial infarction and positively regulates angiogenesis through multiple signaling pathways (45). The endothelial-cell-specific miR-126 is found on chromosome 9 within intron 7 of the epidermal growth factor (EGF)-like-domain, multiple 7 (EGFL7) gene in the human genome. EGFL7 is secreted by endothelium and regulates angiogenesis and encodes an endothelial-cell-enriched growth factor involved in the control of cell migration (46). Blood flow can induce miR-126 to stimulate vascular endothelial growth factor signaling and controlling angiogenesis of aortic arch vessels (47). miR-126 represses the actions of the Sprouty-related protein, SPRED1, and phospho-inositol-3 kinase regulatory subunit 2, both negative regulators of VEGF signals. Lack of miR-126 in mice cause leaky vessels and defects in angio-genesis but does not cause mortality (48, 49).

Antisense-oligonucleotide-mediated knock-down of miR-126 in zebrafish causes complete embryonic lethality owing to the loss of vascular integrity and hemorrhaging (50).

One other miRNA which plays a role in angiogenesis is miR-21. PTEN and Bcl-2, two important signal molecules associated with vascular smooth muscle cells (VSMCs) growth and apoptosis, are the targets of miR-21, through which miR-21 exerts its function (51). Overexpression of this miRNA induces high expression of HIF-1alpha and VEGF, both of which promote angiogenesis. Indeed, cells transfected with miR-21 induced more branching of micro vessels in the Chick Chorioallantoic Membrane (CAM) assay (52).

Apart from their role in angiogenesis and its promotion, miRNAs have other roles in vascular system. VSMCs are able to perform both contractile and synthetic functions, which are associated with changes in morphology, proliferation and migration rates and are characterized by the specific expression of different marker proteins. Several miRNAs, including miR-143 and miR-145 have a demonstrated role in VSMC differentiation (53).

miR-143 and miR-145 encoding genes are highly conserved and lie in close proximity with each other on human chromosome 5 (54, 55). Studies based on northern blotting indicated that the highly conserved miR-143/145 encoding genes are expressed in various mouse tissues and specifically in smooth muscle cells (SMCs) under the control of serum response factor (SRF) and members of the myocardin family of co-activators (31) and target several regulators of actin signalling, including Rho GTPases, sling -shot homologue 2, adducin, cofilin and actin itself (56). According to Elia et al. miR-143 has higher expression in heart than in other organs and is expressed in lung, skeletal muscle, heart, and skin and is most abundant in aorta and fat, where miR-145 is also at its highest expression level (53, 57).

Creating genetic mutation in miR-143/145 in vivo proved that these miRNAs are unessential for smooth muscle specification, but they are required for switching proliferative and contractile VSMC phenotypes to each other. Experiments demons-trated that because of their destabilizing effect on transcripts encoding the repressor of the contractile VSMC phenotype, including klf4, klf5 and Ace, in vivo, mice lacking miR-143/145 display reduced arterial medial thickness, decreased vascular tone and reduced systemic blood pressure during homeostasis (58).

Also, Human Genome Wide Association studies have identified SNPs in the miRNAbinding sites of several RAAS (renin-angiotensin aldosterone system)-associated genes that correlate with a dysregulation of blood pressure (58).The renin-angiotensin aldosterone system is a hormonal cascade that functions to control arterial pressure, tissue perfusion, and extracellular volume (59).

Another mirRNA that we discuss in this review is miR-451. Studies have demonstrated that the highly conserved miR, miR-451, is expressed extensively in endothelial and blood cell lineages during embryogenesis and has distinct functions during in vitro embryonic stem cell differentiation by promoting the differentiation of endothelial and blood cells while blocking the differentiation of cardiomyocytes. This function is achieved at least partially through the down regulation of target Acvr2a and up regulation of Wnt signaling (60). Pharmacological knockdown of miR-451, results in reduced baseline hematocrit levels and impaired erythroid expansion in response to oxidative stress (61-63).

**miRNAs as therapeutic agents**

There are some therapeutic structures and drugs for curative and palliative therapy of the disease, but to the aim of accessing reliable therapy or to postpone the onset of disease, especially for people with heritable coronary artery disease in their pedigree. Genetic engineering technologies are making advances in the field by continually identifying oligonucleotides with higher potencies, particularly interfering RNA (miRNA); more effective cellular targets, typically in signaling pathways; and delivery approaches with the next generation of drug platforms (64). The presence of cell-free miRNAs has been detected in a range of body fluids. The miRNA content of plasma/serum in particular has been proposed as a potential source of novel biomarkers for a number of diseases (65). So, there are many miRNAs which participate in cardiac related diseases and also their changes can be considered as a biomarker for the disease (27-30). The use of miRNAs as biomarkers has greatly increased as a result of the discovery that they are present in the circulating blood. A number of groups have shown that miRNAs can be detected in human serum or plasma, where they are thought to be protected from degradation by being encapsulated in microvesicles or exosomes and/or are bound by RNA-binding proteins such as Ago2 and nucleophosmin (19, 30, 65). These findings can be considered as new strategies for cardiovascular disease therapies.

## Conclusion

microRNAs are responsible for regulating gene expression through translation inhibition or transcriptional degradation. Several miRNAs have been linked to cardiac hypertrophy, myocardial infarction and atherosclerosis. Scientists are now profiling circulating and disease-specific microRNAs in an effort to identify predictive biomarkers and understand disease processes.

Among these miRNAs, some of them were recognized as the key elements in processing of CAD such as miR-126, miR-21, miR-143 and miR-145. Thus, the next researches should be focused on the accuracy of the miRNAs as biomarker in CAD.
